# Progress in Surgery

**Published:** 1896-05-09

**Authors:** 


### PROGRESS IN SURGERY.

### SURGERY OF THE SPINE.

**Lumbar Puncture of the Sub-Arachnoid Space.**—I. In 1891 Quincke first suggested tapping the cerebro-spinal fluid in the lumbar region as a diagnostic and therapeutic measure in cases of increased intra-cranial tension from various causes. Since that time several hundred cases have been recorded in which this procedure has been employed. In an elaborate and most instructive paper Jacoby[1] discusses the subject in its present-day aspect. The procedure, he says, is based upon two facts: (1) That the sub-arachnoid spaces of the brain and cord communicate with each other, and with the ventricles of the brain; and (2) that the spinal cord reaches in the adult to the second, and in children to the third, lumbar vertebra; that therefore a needle passed into the third or fourth inter-laminar space does not strike the cord, but passes among the floating nerve-roots of the cauda.

The object of the operation may be twofold: (1) To evacuate fluid for relief of tension; or (2) to obtain fluid for diagnostic examination.

In describing the technique of the operation, he emphasises the importance of entering through the second or third lumbar interspace, and he suggests a useful landmark in determining these spaces. A line joining the highest points of the iliac crests passes across the centre of the fourth lumbar spine, and it is a simple matter to count from this. Although an anæsthetic is not indispensable, it is to be recommended in most cases. The patient is placed on his side, the head and shoulders well bent forward, and the thighs flexed on the abdomen. The trochar is passed in about two to six centimetres, according to the nutrition of the patient. The quantity of fluid withdrawn varies greatly, depending on the object desired and the amount of fluid present, &c. Aspiration is to be avoided in the great majority of cases, as it greatly increases the pain of the operation, which is otherwise almost nil. The complications likely to be encountered in performing the operation are: (1) The needle getting blocked by a floating nerve root; (2) bleeding from puncture of a spinal vein or from striking bone; (3) puncture of a nerve-root or cord, giving rise to spasms. The risk of septic infection with moderate care is very small. Stadelmann, however, points out the risk of a localised meningitis being converted into a general one by this procedure. No permanent drainage is kept up, the cannula being withdrawn, and the puncture sealed with collodion, and covered with wool and a bandage.

As regards the *therapeutic value* of spinal puncture, results are not encouraging. Hydrocephalus (acute and chronic), meningitis (serous, tubercular, alcoholic, and cerebro-spinal), encephalo-malachia, chronic myelitis, tabes, sub-dural spinal hæmorrhage, have all been treated by means of lumbar puncture, without other result than transient relief of certain symptoms, such as headache, epileptiform convulsions, spasms, &c. It is worthy of note that in cases of brain tumours the operation is usually followed by a very great exacerbation of pain, which lasts for about a quarter of an hour, and then gradually passes off. In compression myelitis and meningitis cures have been alleged. In tubercular meningitis nothing but the slightest amelioration, and even that rarely, has been observed.

The *diagnostic value* of spinal puncture is much more definite, and it is gradually assuming an important place amongst the means at our disposal of recognising the nature of intra-cranial and spinal affections. Quincke asserts that 1 to 2 per cent. of albumin points to an acute exudation, *i.e.*, to fresh inflammation, and others confirm this. In cases of brain tumour the albumin is much less (0·4 to 0·8 per cent.). Lichtheim found sugar in cases of brain tumour, rarely in other conditions; and Jacoby found it in three cases of acute mania. But by far the most important point, from the diagnostic point of view, is the recognition in the fluid of micro-organisms—cocci in simple meningitis, and the tubercle bacillus in the tubercular variety. Although a difficult matter in some cases, positive results are almost always forthcoming. Jacoby reports a case in which tubercle bacilli were detected in the fluid before grave symptoms became manifest. Blood in the fluid is of doubtful diagnostic value.

II. Furbringer[2] refers to some cases in which no fluid was obtained on making a spinal puncture, although the needle had entered the dural space. In one instance, a case of tubercular cerebro-spinal meningitis, the space was found, *post-mortem*, to be distended by a semi-solid œdematous material with practically no fluid.

III. The same writer in a more recent paper[3] draws special attention to the fatal cases of lumbar puncture. Out of 87 cases reported by him, five have died, two within a couple of hours, one six hours, and two within forty-eight hours of the operation. Whether death was to be directly attributed to the puncture was in all the cases difficult to determine. Three of the patients suffered from intra-cranial tumour, and two had uræmia. Lichtheim also had a fatal case in cerebellar tumour. Furbringer strongly opposes the further treatment of intra-cranial, and especially cerebellar, tumour by this method.

**Injuries to the Spine.**—I. In a clinical lecture Mr. H. W. Page[4] discusses some points with regard to fracture dislocation of the spine, more especially in connection with the condition of the reflexes as a diagnostic sign, and as an indication for treatment. He takes it as an accepted fact that the cord is injured in fracture-dislocation either by displacement of vertebræ, transient or abiding, or by the bend to which it has been exposed in over-bending of the column. The indications of grave injury are that the patient is paralysed both as to motion and sensation in the parts below from the moment of the accident, and the level of the lesion is diagnosed by observing carefully the area of anæsthesia and the paralysed muscles. The condition of the reflexes is also of great importance. All seem to agree now that in complete transverse division of the cord the deep reflexes are instantly and permanently abolished; but opinions vary as to the state of the superficial reflexes. Bowlby found in some cases that the superficial reflexes persisted, but Thorburn is of opinion that this is due to some part of the cord not having been completely divided. In nineteen cases of his own, both deep and superficial reflexes were absolutely lost. He sums up: (1) Shock is not the cause of early loss of reflexes in spinal injury; (2) when the lesion causes complete paralysis and anæsthesia the deep reflexes are always lost; (3) when motor or sensory power, or both, return in even a slight degree, these reflexes also return; (4) when no such recovery occurs the deep reflexes remain absent for periods of practically indefinite duration; (5) when the lesion does not cause complete paralysis and anæsthesia, the deep reflexes remain in either a normal or an exaggerated form; (6) but if the motor and sensory symptoms afterwards become complete, such reflexes disappear; (7) the superficial reflexes do not conform absolutely to these rules. In nearly all cases, both of complete and partial lesions, these disappear and remain absent, the "plantar reflex" only existing. Reference is made to a very full digest of this subject by Dr. Reynolds in the spring number of *Brain*, 1895, and the paper contains full clinical notes of three of the writer's own cases.

II. Dr. A. C. Haven[5] reports an unusual case of gunshot wound of the cervical spine. The ball entered the right side of the neck, on a level with the upper border of the thyroid cartilage, and was removed from the opposite shoulder midway between the spine and the acromion, on the upper border of the trapezius muscle. The damage done was confined to the spinal canal, where a clot pressed on the left side of the cord, producing paralysis and anæsthesia on that side. Sensation was also affected on the opposite side, showing pressure on both posterior roots. Four months after injury he was almost well.

**Laminectomy in Potts' Disease and Spinal Injury.**—The value of this operation is one of the most disputed points in spinal surgery. Stemen,[6] in reporting a series of cases in which he has trephined the spine, says that, in face of the results obtained by himself and others, "he cannot conceive how any man can oppose the operation in cases where there is clearly compression of the cord. He refers specially to cases of injury. On the other hand Calot,[7] writing of Potts' disease, holds that our present knowledge does not justify operations in cases of paralysis due to Potts' disease, as most of these cases will recover with prolonged rest. Nineteen out of twenty of his own cases recovered after prolonged rest. Dr. Sinclair White, of Sheffield,[8] reports a successful case of laminectomy for paraplegia due to spinal caries in a child eight years of age. This surgeon believes that the operation should be reserved for cases which have resisted long-continued extension and counter-extension, or where this method is inapplicable, each case being decided on its own merits. Dr. Ménard[9] has had several successful cases. He is inclined to believe that the operations which are followed by relief of paralysis are those in which an abscess is found and drained.

**Spina Bifida.**—Dr. Marshall, of Nottingham,[10] reports a case of lumbar spina bifida, successfully treated by ligature of the sac, the reason for operating being the rapid growth of the swelling. Edwards[11] recently had an opportunity of examining a case of spina bifida in the cervical region. At the birth of the child a swelling about the size of a tangerine orange was observed on the back of the neck. The child only lived twelve hours, during which time it had several fits. On *post-mortem* examination the tumour was found to communicate with the interior of the skull by passing through the foramen magnum, and an opening in the neural arch of the atlas. The foramen magnum and the other neural arches were normal.

**Tumour of the Spinal Canal.**—Kümmell, of Hamburg,[12] recently removed a sarcoma from the spinal canal which had caused pressure symptoms referable to the upper dorsal region of the cord. The points of special interest in the case are: (1) That the tumour was secondary to a rectal sarcoma removed some time before; (2) immediately on removing the tumour from the canal, the cord, which was considerably pressed upon, began to increase in size and to resume its normal shape; (3) the great improvement following the operation; and (4) the bad prognosis as to recurrence in other organs.

[1] N. Y. Med. Journ., Dec. 28th, 1895. [2] Deutsh. Med. Woch., Nov. 7th, 1895. [3] Centralbl. f. Inn. Med., Jan. 4th, 1896. [4] Lancet, Feb. 8th, 1896. [5] Med. Record, Feb. 15th, 1896. [6] State Med. Journ., Nov., 1895. [7] Med. Record, Dec., 1895. [8] Quart. Med. Journ., Jan., 1896. [9] Rev. d'Orthopedic, March, 1895. [10] B. M. J., Jan. 18th, 1896. [11] B. M. J., Nov. 30th, 1895. [12] Beiträge zum Centralbl. f. Chirurg., 1895.

### DISEASE OF THE UPPER AIR PASSAGES.

(*Continued from page 78.*)

**Supperating Ethmoiditis.**—R. C. Myles[6] records a case of frontal and posterior ethmoidal abscess with polypoid degeneration, in which there had been a history of extreme suffering for twelve years, the principal symptoms being headaches, a nervous and unsettled mental condition, sleeplessness, nasal catarrh, a tickling sensation in the throat, and a great deal of sputa, mostly from the bronchi. Chiari[7] treats frontal sinusitis by irrigations through a thin canula introduced into the excretory duct of the frontal sinus, with or without removal of the anterior end of the middle turbinate. He had cured by one single injection two cases of acute catarrh of the frontal sinuses with intense cephalalgia, and one case of chronic mucous retention; and two cases of chronic suppurative catarrh of the frontal sinuses, with frontal pains, have also been cured by repeated injections. Ziem,[8] of Dantzic, condemns the employment of the galvano-cautery to the anterior end of the middle turbinate for the purpose of restoring the patency of the fronto-nasal duct, and remarks that the anterior end of the middle turbinate is also too frequently removed; free scarification in this region would certainly be less harmful, and perhaps as effective. He agrees with Moure as to the danger of making a false passage and even entering the cranial cavity in attempting the passage of a probe into the fronto-nasal duct, and reminds us of one case of such an accident, reported to the Congress of Southern German Laryngologists held at Heidelberg in 1895. He also points out the difficulties and objections to Schäffer's method of breaking through the inferior wall of the sinus from the nose. He advises, and has in one case used without an anæsthetic, a dental drill for entering the frontal sinus externally, the integuments having been incised and drawn aside, so as to get an exploratory irrigation.

If pus is present it is necessary to adopt one of the following procedures: (1) To widen the opening to one or one and a half centimetres by means of a trephine fitted to the dental engine; (2) if the sinus extends considerably towards the temple, which may be determined by means of a bent probe, a second opening, three or four millimetres broad, may be made as near the temple as possible, in order to allow of a more thorough washing of the cavity; (3) to remove the orbital wall of the sinus by Jansen's method; (4) to remove the entire anterior wall of the sinus, according to the method hitherto practised in surgery, and most recently in a large number of cases by Kuhnt; (5) to apply Czerny's method, which consists in making an osteoplastic resection of the anterior wall of the sinus. Kuhnt's method gives very rapid and satisfactory results, and Kuhnt claims with but little resulting deformity.

In treating cases of suppurating ethmoiditis Bryan first removes the cap of the middle turbinated body, either with the snare or the electric burr, and then continues opening the individual cells with a sharp curette, inasmuch as this is a dangerous region. If the deeper, or fronto-ethmoidal, cells are involved, then we should not attempt to relieve them through the nose (although it was done successfully in one of Bryan's cases) as the operation is not unattended with danger, but the case should be treated as one of frontal sinus abscess, of which this condition is a very frequent complication. The same object is to be sought in the treatment of abscess of the frontal sinuses as in the other neighbouring cavities of the nose—that is, to evacuate the pus and establish free drainage. In a few cases this can be accomplished by removing any hypertrophy, polypi, or anything within the nose that serves to obstruct the fronto-nasal duct, when, if this duct is pervious, free drainage can be maintained and the patient eventually recover. If, however, the duct should be blocked, then an attempt should be made to pass a probe into it so that its permeability can be re-established. Sounding the fronto-nasal duct is by no means an easy procedure, and when possible it is more applicable in cases of mucocele than in cases of suppurative inflammation, and prevents the former painful condition from being converted into the much more serious affection of abscess of the frontal sinus. But in the majority of cases of abscess, if of long standing, it will be found necessary to make an artificial opening.

Chiari's[9] treatment of ethmoidal empyema necrosis consists in removing the necrosed bone, opening the cells filled with pus (with or without curetting), in removing the small osseous lamellæ and hypertrophied mucous out-growths, and without ever producing great reaction. Suppuraton then ceases in his experience, and irrigations completely cure, or at least notably improve the condition. He shares Grünwald's opinion that there exist ethmoidal-suppuration necroses, which are neither syphilitic nor tubercular, none of his patients suffering from these affections; but he is still strongly opposed to the idea that recurrent nasal polypi and ozæna are commonly caused by ethmoidal suppurations, caries, and necrosis, for in 61 cases of polypi and in 128 cases of ozæna he has never met with these ethmoidal diseases in spite of the most careful examinations.

**Sphenoidal Sinus Abscess.** — Bryan[10] accords with general experience when he remarks that suppurative disease of the sphenoidal sinus occurs much more rarely than it does in any of the other accessory cavities. While it does occur as an independent affection, it is much more frequently observed as a complication of abscess of the ethmoidal sinus, where the thin bony partition between the posterior cells and the sphenoidal sinus is broken down and their contents discharged into the latter cavity. The treatment of these cases will occasionally be found tedious and discouraging, but if care has been taken to establish free drainage, and the antiseptic applications are thoroughly applied, the majority of patients will recover.

F. Bosworth[11] has had a very large experience in suppurative ethmoidal sinusitis, and his remarks are worthy of note when he says that while he does not regard ethmoid disease as dangerous to life, yet the possibility of an invasion of the sphenoidal sinuses is something always to be considered in giving advice in these cases; and, furthermore, whereas the literature of the last four years has contained reports of a large number of cases of sphenoidal disease, he is led to think, in reading the detailed history of many of these cases, that possibly some of them were cases of ethmoid disease reported as sphenoidal. His own records show reports of 150 cases or more of ethmoid diseases which, as a rule, have yielded more or less satisfactorily to treatment, and in all his experience he has seen but two cases of sphenoidal disease, and both terminated fatally. He records one case where ethmoid disease existed several years, with only offensive discharge and occasional headaches, when he was seized with intense and excruciating headaches, which seemed neuralgic in character, and was confined largely to the left side of the face and head. There was no psychical disturbance, photophobia, and no fever, and the pain was constant. A few days later, after temporary relief by opening the posterior ethmoidal cells, the patient died with symptoms of brain abscess. Bosworth was of opinion that this was an instance of ethmoidal abscess rupturing into the sphenoidal sinus with onset of the acute symptoms, and terminating with secondary intra-cranial abscess and death. He remarked that in one case of his a probe passed to the posterior wall of the sphenoidal sinus measured 6¼ inches from the tip of the nose. A probe inserted from 2½ to 3¼ inches might carry it into the ethmoidal cells, but not into the sphenoidal sinus. Fletcher Ingals,[12] with the aid of a dental instrument, working at right angles behind the palate, has succeeded in perforating the sphenoidal cells on their under surface in three cases of sphenoidal abscess. Gleitsmann[13] always opened the sphenoidal sinus with a strong probe, and not with a trephine. He found the anterior wall soft in chronic cases. In one, severe secondary hæmorrhage, which he could not explain, supervened. Mulhall[14] agreed that diseases of the sphenoidal sinus, apart from the ethmoidal cavities, are certainly very rare; he had had many cases of ethmoidal abscess, and had operated very freely with the burr, and had had no serious results. In one case only had he discovered disease of the sphenoidal sinus uncomplicated with disease of the surrounding sinus, and this had occurred in a young woman with a history of a headache which had lasted sixteen years. On passing a probe bare bone had been found, indicating disease of the sphenoidal sinus. The opening was enlarged with a curette, and kept open for a considerable time. Chiari has sounded the sphenoidal sinus three times; of course, in cases where a purulent collection of the olfactory cleft or of the naso-pharyngeal cavity raised a suspicion of an affection of these sinuses. Hajek[15] considers that we ought to make only a small opening into the sphenoidal sinus; nor should the mucous membrane of the cavity be scraped or cauterised, since these are two severe operations never indicated by the small amount of suffering which catarrh of the sphenoidal sinus provokes. Herzfeld[16] recommends opening the cavity of the sphenoidal sinus by introducing a bistoury immediately below the lower edge of the middle turbinated, and, if needful, the opening may be enlarged. Ziem[17] is of opinion that the value of injections through the *ostium sphenoidale* by means of the hollow sound recommended by Lichtwitz appears somewhat doubtful, for the return of the injected fluid cannot be looked for with certainty. When the diagnosis has been made with some degree of probability, the establishment of a counter opening is to be preferred. This is best and most expeditiously done by means of the drilling machine fitted with long points. He refers to the danger of endeavouring to open the sinus by means of a drill introduced at the level of the middle turbinated, for we may encounter the basilar process and not the sinus by proceeding thus. He recommends that in patients whose throats are roomy and easily palpated, the instrument be passed along the inferior meatus, then, with the finger introduced into the naso-pharynx, it may be guided into position.

[6] Journ of Laryng., March, 1896, p. 144. [7] Rep. of Vienna Lar. Soc., Journ. of Laryng., Jan., 1896. [8] Journ. of Lar., Dec., 1895. [9] Loc. cit. [10] Loc. cit. [11] Ibid. [12] Loc. cit. [13] Loc. cit. [14] Loc. cit. [15] Ibid. [16] Loc. cit, [17] Loc. cit.

